# Acute Antioxidant Response to Two Types of Exercises: 2000 M Run vs. Burpee Test

**DOI:** 10.3390/antiox13020144

**Published:** 2024-01-24

**Authors:** Lucrecia Carrera-Quintanar, Lorena Funes, María Herranz-López, Néstor Vicente-Salar, Juan Mielgo-Ayuso, Manuel Moya-Ramón, Antoni Pons, Vicente Micol, Enrique Roche

**Affiliations:** 1 Doctorate in Translational Nutrition Sciences (DCNT) University Center of Health Sciences (CUCS), University of Guadalajara (UDG), Guadalajara 44340, Mexico; lucrecia.carrera@academicos.udg.mx; 2 Institute of Research, Development and Innovation in Healthcare Biotechnology of Elche (IDiBE), Miguel Hernández University (UMH), 03202 Elche, Spain; innovation@vitalgrana.com (L.F.); mherranz@umh.es (M.H.-L.); 3 Department of Applied Biology-Nutrition, Institute of Bioengineering, Miguel Hernández University (UMH), 03202 Elche, Spain; nvicente@umh.es; 4 Alicante Institute for Health and Biomedical Research (ISABIAL), 03010 Alicante, Spain; 5 Department of Health Sciences, Faculty of Health Sciences, University of Burgos, 09001 Burgos, Spain; jfmielgo@ubu.es; 6 Department of Sport Sciences, Sports Research Center, Miguel Hernández University (UMH), 03202 Elche, Spain; mmoya@umh.es; 7 Research Group on Community Nutrition and Oxidative Stress, University of Balearic Islands, 07122 Palma de Mallorca, Spain; antonipons@uib.es; 8 CIBER Physiopathology of Obesity and Nutrition/Fisiopatología de la Obesidad y la Nutrición (CIBEROBN), Instituto de Salud Carlos III (ISCIII), 28029 Madrid, Spain

**Keywords:** aerobic exercise, anaerobic exercise, antioxidant enzymes, oxidative stress, sports recovery

## Abstract

Physical activity results in oxidative stress, as evidenced by the increased production of reactive oxygen, nitrogen species, and inflammatory mediators. The management of these components is instrumental for antioxidant adaptation to exercise and post-exercise recovery. Therefore, the present report aims to study the antioxidant response to two types of exercise (a 2000 m run and a burpee test) in healthy volunteers after a long period of inactivity (1–2 months). Antioxidant enzyme activities and oxidative stress markers (protein carbonyls and malondialdehyde content) were measured in neutrophils, peripheral blood mononuclear cells, and plasma. These parameters were determined under basal conditions and immediately post-exercise. Compared to those in basal state, neutrophil superoxide dismutase (28.3 vs. 22.9 pkat/10^9^ cells), glutathione peroxidase (147.5 vs. 120.1 nkat/10^9^ cells), and catalase (106.3 vs. 57.9 k/10^9^ cells) were activated significantly (*p* < 0.05) after the burpee test. Peripheral blood mononuclear cells exhibited only significant (*p* < 0.05) catalase activation (113.6 vs. 89.4 k/10^9^ cells) after the burpee test. Other enzymes, such as glutathione reductase and myeloperoxidase, tended to increase post-exercise, although the differences from baseline were not significant. Finally, compared to basal conditions, the protein carbonyl (24.5 vs. 14.5 mmol/L) and malondialdehyde (39.6 vs. 18.3 mmol/L) contents increased significantly (*p* < 0.05) in neutrophils and in plasma (115.1 vs. 97.8 and 130.2 vs. 123.4 μmol/L, respectively) after the burpee test. In conclusion, high-intensity exercise seems to induce immediate oxidative stress in inactive individuals, and the acute antioxidant response was slightly greater after the burpee test than after the 2000 m run. Glutathione-dependent antioxidant systems are activated immediately as protective mechanisms.

## 1. Introduction

Physical activity favors oxidative stress, as evidenced by the increase in the production of reactive oxygen and nitrogen species (RONS) and inflammatory mediators [1]. In addition to exerting deleterious effects, RONS generated during exercise are key factors for skeletal muscle adaptation to exercise as intracellular signaling molecules in the activation of genes that encode antioxidant enzymes. These genes are regulated by Nrf2, a master transcriptional regulator responsible for cellular redox homeostasis [2,3]. The main antioxidant enzymes include catalase (CAT), superoxide dismutase (SOD), glutathione peroxidase (GPX), and glutathione reductase (GRD) [4]. Their activation in those genes is instrumental for the optimal adaptation of muscle tissue to exercise, reducing long-term oxidative stress associated with sport activity and improving the health status of the individual [5,6]. This particular mechanism is known as exercise-induced hormesis: low or controlled stress favors adaptation, but high or uncontrolled stress causes damage [7,8,9]. The conditions of high stress (strenuous exercise) can overwhelm endogenous antioxidant capacity, making antioxidant supplementation necessary [10,11,12].

Exercise-induced hormesis depends on several variables, such as the type of exercise, intensity, and training status of the individual [13]. These variables are interconnected, making it difficult to reach clear conclusions by comparing different studies. In this context and regarding the type of exercise, both aerobic and anaerobic exercise seem to increase oxidative stress and the antioxidant response to different extents [14,15,16]. Compared to aerobic exercise, anaerobic exercise generates a greater amount of RONS, and anaerobic–alactic efforts produce more oxidative stress than anaerobic–lactic efforts. However, recovery is faster after anaerobic exercise than after aerobic exercise, which can last several hours due to muscle glycogen depletion [17]. In addition, protein and glutathione oxidation increase following anaerobic exercise, but no significant increases in malondialdehyde (MDA) (a marker of lipid peroxidation) are reported after the execution of either type of exercise [18]. In addition, resistance training, which is mainly fueled by anaerobic metabolism [19], promotes the generation of RONS, which can activate endogenous antioxidant responses [20]. Nevertheless, the resistance training modality seems to play a role in this response. Similarly, high-intensity interval training (HIIT) seems to be more efficient in reducing oxidative damage and increasing antioxidant activity [21] than classical resistance training [19].

Intensity is another variable to consider. In this context, oxidative damage does not depend on the intensity of the damage but rather on the training status of the individual. Trained individuals take longer to reach exhaustion than untrained individuals performing the same type of exercise [22]. Therefore, trained individuals have better antioxidant adaptation and exhibit less oxidative stress [23]. Nevertheless, a certain intensity is necessary to trigger the initial adaptation to oxidative stress and the activation of the antioxidant response [2]. However, after this adaptation period, high-intensity exercise can overwhelm the antioxidant capacity, which could be mitigated by antioxidant supplementation [22]. Moreover, recent reports seem to indicate that low or moderate intensities can induce oxidative stress, indicating that exercise volume (intensity × time) could be the key factor in inducing oxidative stress [13]. Similarly, high-intensity exercise seems to increase the total antioxidant capacity compared to low- and moderate-intensity exercises according to treadmill tests [24].

Little information is available regarding antioxidant responses in inactive individuals who do not display an initial adaptation to vigorous effort. High-intensity routines result in increased markers of oxidative damage in skeletal muscle and blood. However, when high-intensity routines are performed chronically, antioxidant adaptations appear to attenuate the oxidative stress response [25]. Nevertheless, high-intensity exercise comprises a wide variety of routines and body systems, including cardiorespiratory and muscular strength efforts. It has been suggested that different routines result in oxidative stress but elicit different antioxidant responses [10]. For instance, anaerobic eccentric exercise can cause more muscle damage [26] than concentric exercise [27]. Therefore, antioxidant adaptation in high-intensity exercise conditions is less clear.

The objective of the present study was to study the acute antioxidant response to two bouts of exercise: a 2000 m run and a burpee test. The first test elicits substantial involvement of the cardiovascular system and lower body musculature, while the burpee test incorporates the whole-body neuromuscular system to a greater extent.

## 2. Materials and Methods

### 2.1. Participants and Intervention Protocol

Twenty male volunteers (20–22 years old) were selected from the Sports Sciences and Physical Activity students at University Miguel Hernández of Elche (Spain) before starting the school year. Body composition parameters were as follows: body mass index (BMI) = 22.1 ± 1.9 kg/m^2^); muscle mass = 42.4 ± 3.1%; and fat mass = 13.5 ± 2.4%. Volunteers performed recreational physical activities regularly but did not participate in organized training programs. The participants were recruited after a period of inactivity (1–2 months) during the summer season to provide a homogeneous physical condition among them. The inclusion criteria were as follows: did not perform any training protocol before (i.e., during the summer break) and during the intervention period (1 week); did not present any chronic disease; did not smoke or drink alcoholic beverages; followed the diet designed by the nutritionist of the research team during the intervention; completed a 2000 m run in less than 8 min; and completed 30 repeations or more during the burpee test. The exclusion criteria were as follows: the consumption of antioxidants or any type of ergogenic supplements during the intervention; the consumption of anti-inflammatory drugs or any type of medicine; or muscle damage due to unexpected events at the time of the study. Participants were informed about the objectives of the intervention and voluntarily signed a written consent form. The study was conducted in accordance with the Helsinki Declaration for research on human beings and was approved by the Ethics Committee of University of Balearic Islands with reference to IB 994/08 PI.

On Day 1, participants meeting the inclusion criteria were selected to undergo an initial anthropometric evaluation and blood extraction in a certified laboratory. Subjects with similar body compositions were recruited in order to have a homogeneous sample. The next day (Day 2), the 2000 m running test was performed. All participants (*n* = 20) performed the test in less than 8 min (range: 7:15–7:54 min). Participants were assessed after 5 days (Day 5) for the burpee test. Only 18 participants were capable of performing 30 or more repetitions (range: 30–32 repetitions). The 2 volunteers who performed fewer than 30 repetitions were excluded from the data analysis. Participants wore Polar RS-800 accelerometers (Barcelona, Spain) during the tests to monitor heart rate changes in beats per min (bpm). Blood samples were obtained by a 3-nurse team immediately after each test (2000 m run and the burpee test) during a 5–7 min time interval. Participants did not perform sports or training activities during the period between the tests (Days 2–5). The methodology used, the interpretation of data, and the working concepts were explained and discussed during classes throughout the academic course.

The physical and health status of each participant was determined according to anthropometric criteria and blood analysis, respectively, at the beginning of the intervention (Day 1). Anthropometry was performed according to the International Society for Advancement of Kinanthropometry (ISAK) recommendations [28]. Blood samples (10–12 mL) were obtained in EDTA vacutainers from the antecubital vein after overnight fasting for Day 1. Post-exercise blood extractions from Days 2 and 5 were not performed in the fasting state to replicate the conditions under which high-intensity physical activity is typically undertaken and with the aim of optimizing the participants’ performance. Circulating parameters on Day 1 were determined according to standard laboratory procedures [29]. These measurements included circulating cell counts, lipid profile (triglycerides, total cholesterol, low-density lipoprotein, or LDL, and high-density lipoprotein, or HDL, glucose, creatinine, urea, and uric acid levels, and hepatic and muscle markers such as alanine aminotransferase, aspartate aminotransferase, and γ-glutamyltransferase. The muscle markers creatine kinase (CK) and lactate were measured at Days 1, 2, and 5 to monitor post-exercise muscle damage and exercise performance, respectively.

Caloric expenditure was calculated taking into account the resting metabolic rate (according to the Harris–Benedict equation), the thermal effect of food (estimated as 8.5% of the total caloric expenditure), and physical activity expenditure estimated from [30]. The Dietsource program (Novartis, Barcelona, Spain) was used for diet design. Daily intakes were estimated at 2000–2200 kcal broken down as 6–7, 1.2–1.5, and 1 g/kg/day of carbohydrates, proteins, and lipids, respectively. The dietary daily food antioxidant intake was ~125 mg of vitamin C and ~5 mg of vitamin E. Breakfast was consumed 1–2 h before each test and was rich in carbohydrates. After each exercise, participants were advised to rehydrate with an isotonic drink and consume a piece of fruit. The diet was designed by a nutrition professional, and email contact was provided to participants to resolve doubts regarding diet compliance.

### 2.2. Blood Cell Purification

Neutrophils, PBMCs (peripheral blood mononuclear cells), and plasma were obtained as indicated previously immediately after each extraction [11,31,32]. Briefly, a blood aliquot was carefully placed in a Corning tube on Ficoll (GE Healthcare, Stockholm, Sweden) at a ratio of 1.5:1 vol and centrifuged for 30 min at 900× *g* and 4 °C. Pellets containing erythrocytes and neutrophils were incubated at 4 °C with 0.15 M ammonium chloride to lyse erythrocytes. The final suspension was centrifuged for 15 min at 750× *g* and 4 °C. The supernatant was discarded, and the bottom phase containing neutrophils was washed with ammonium chloride and then with a phosphate-buffered saline (PBS) solution at pH 7.4. The intermediate layer obtained from the Ficoll gradient containing PBMCs was carefully removed, washed twice with PBS, and centrifuged for 10 min at 1000× *g* and 4 °C. The obtained neutrophils and PBMCs were lysed with double-distilled water to measure antioxidant enzymatic activities and the oxidative stress markers MDA and protein carbonyls. Plasma was obtained from the supernatant of another blood aliquot after centrifugation at 1000× *g* for 15 min at 4 °C and stored at −80 °C.

### 2.3. Determination of Antioxidant Enzymatic Activities and Oxidative Stress Markers

Antioxidant activities and oxidative stress markers were determined the following week, immediately after completing the intervention, in the frozen samples. Neutrophil and PBMC CAT, GPX, and GRD SOD activities were measured on a microplate reader (SPECTROstar Omega, BMG LabTech GmbH, Offenburg, Germany) at 37 °C, as indicated previously [31]. Myeloperoxidase (MPO) was selected as an inflammatory marker, and its activity was determined by guaiacol oxidation as described previously [31]. Protein carbonyl levels were determined in neutrophils, PBMCs, and plasma, as described previously [32]. MDA was determined by HPLC, as described previously [31].

### 2.4. Statistical Analysis

Statistical analysis was carried out using SPSS-26 software for Windows. The data follow a normal distribution according to the Shapiro–Wilk test. The repeated measures analysis of variance (ANOVA) was used. In terms of variables, the dependent variable corresponds to the enzyme activity/blood marker. The independent variable (time or condition) has three levels: before exercise (baseline condition), after the 2000 m test, and after the burpee test. The significance of multiple comparisons was tested by the Bonferroni test. The results are expressed as the mean ± SEM (standard error of the mean). Values with a *p* < 0.05 were considered to indicate statistical significance.

## 3. Results

Blood analysis was performed to check the health status of each participant. Circulating cell counts, lipid profile (triglycerides, total cholesterol, LDL, and HDL), glucose, creatinine, urea, and uric acid levels were within the healthy range [29] (Table 1). Hepatic and muscle marker levels, such as alanine aminotransferase, aspartate aminotransferase and γ-glutamyltransferase were also within the healthy range [29] (Table 1), confirming that the participants were in good health. Regarding body composition, participants displayed normal weight (BMI = 20–25 kg/m^2^) and not excessive amounts of fat mass (<15%).

The main changes from baseline after the execution of both exercise tests are indicated in Table 2.

**Figure 1:**
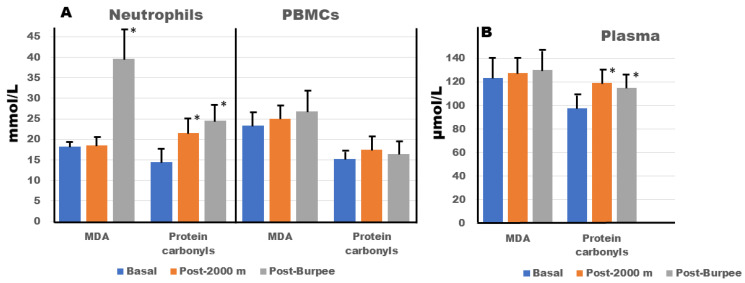
Oxidative stress markers in circulating cells (**A**) and plasma (**B**). * Significant differences (*p* < 0.05) compared to basal conditions. Intracellular malondialdehyde (MDA) and protein carbonyl concentrations were calculated assuming a value of 300 µL/10^9^ cells.

To determine the presence of oxidative stress after exercise execution, we measured the MDA and protein carbonyl levels in neutrophils, PBMCs (Figure 1A), and plasma (Figure 1B). The results indicate that, compared with the basal condition, the MDA concentration increased significantly (*p* < 0.05), but only in neutrophils after the burpee test. On the other hand, plasma protein carbonyl levels were significantly (*p* < 0.05) greater after the 2000 m and burpee tests than at baseline. No significant changes in MDA and protein carbonyl levels were observed in the PBMCs immediately after exercise, although these levels tended to increase.

As shown in Table 3, two antioxidant enzymes were activated immediately and significantly (*p* < 0.05) after both exercise bouts in neutrophils. The activities corresponded to SOD and GPX. SOD is an antioxidant enzyme that catalyzes the dismutation of superoxide radicals (O_2_^−^) into oxygen (O_2_) and hydrogen peroxide (H_2_O_2_) [33]. This latter product is eliminated by GPX activity, which reduces H_2_O_2_ to H_2_O [34]. CAT is an antioxidant enzyme that catalyzes the dismutation of H_2_O_2_ to O_2_ and H_2_O [35]. The enzyme was significantly (*p* < 0.05) activated in neutrophils after the burpee test but was maintained near basal values after the 2000 m run. Regarding other enzymes, GRD is responsible for restoring the glutathione (GSH) levels consumed during the GPX reaction [36]. To this end, GRD needs reduction potential from NADPH provided by the pentose pathway [36]. Neutrophil GRD activity did not significantly increase after either bout of exercise.

The inflammatory response, together with oxidative stress, is a key component associated with exercise-induced muscle damage [37]. In this context, neutrophil MPO activity was determined immediately after each exercise test. The differences in MPO activity with respect to MPO activity under basal conditions were not significant. MPO seems to be activated for several hours post-exercise, as we have documented previously [12]. Furthermore, the 2000 m run and the burpee test significantly (*p* < 0.05) increased CAT activity in PBMCs, which was different from the modest effect of the 2000 m run on CAT activity in neutrophils.

## 4. Discussion

The objective of the present report was to study the acute antioxidant response after two types of exercise: a 2000 m run and a burpee test. The results obtained in the present study indicated that neutrophil SOD together with GPX seemed to constitute the first line of defense against oxidative stress in detrained individuals [33,34]. Therefore, the antioxidant response seemed to be entirely dependent on the enzyme activity that volunteers displayed under basal conditions, immediately after a long period of inactivity. Longer training protocols can activate genes encoding antioxidant enzymes [11]. Therefore, the analysis of the antioxidant response in trained individuals could be more complicated. For this reason, in the present report, we minimized this variable by selecting healthy individuals who were inactive for a period of at least 30 days. The selected individuals were considered to be healthy according to blood parameters. This selection criterion avoids the occurrence of oxidative stress due to certain pathologies. Therefore, the selected population consisted of healthy young men. Additional studies on elderly individuals and women are necessary to complement the obtained data.

In addition, participants followed the same diet to standardize the inclusion of exogenous antioxidant compounds and avoid dietary imbalances that could generate oxidative stress. Nevertheless, it could be interesting to study the first antioxidant response to an intense exercise bout in inactive individuals who do not follow an appropriate dietary pattern.

### 4.1. Presence of Oxidative Stress Post-exercise

Although different technologies can be used to determine oxidative stress [38], we have focused on two markers of main cellular macromolecules: carbonyls for proteins and MDA for lipids. In this context, following an aerobic bout of exercise, approximately 5–10% of intracellular proteins can be oxidized. The presence of carbonyl groups can promote protein turnover through the proteasome system. The removal of oxidized proteins seems to be the first step in maintaining cellular homeostasis, as oxidized proteins function less efficiently than non-oxidized proteins. Therefore, activation of the proteasome system after endurance exercise is an initial step in the adaptative response [17]. The results from the present report indicate that proteins are prone to undergoing oxidative stress modifications, favoring the production of MDA, a final reactive product of lipid peroxidation [39,40]. Although the molecular mechanism needs to be investigated, we suggest that muscle microtrauma, as indicated by an increase in plasma CK levels, may expose membrane lipids to free radicals. This results in the production of MDA [40,41], which subsequently alters proteins. In addition, the 2000 m run is associated with cardiovascular stress and less muscle contribution, while the burpee test requires the contribution of many muscle components in addition to the cardiovascular system. This fact explains why the burpee test results in more oxidative stress and requires the combined participation of SOD, GPX, and CAT to provide a more complete antioxidant response. Additional research is necessary to test this hypothesis.

### 4.2. Post-Exercise Antioxidant Response

In this context, SOD is the specific antioxidant defense against superoxide radicals, the first type of free radical generated during intracellular oxidative events, and is generated in the mitochondrial electron transport chain resulting from exercise [33]. The product from the SOD reaction is H_2_O_2_, a more stable compound that still has a strong pro-oxidant potential [42]. For this reason, SOD must work in coordination with GPX activity, the enzyme that eliminates H_2_O_2_, resulting in H_2_O production [36]. Therefore, the activation of both enzymes in neutrophils seems to follow an efficient antioxidant defense pattern. Nevertheless, GPX requires the consumption of intracellular GSH. GSH can be replenished through GRD activity, which also requires the cell reduction potential of NADPH [43]. In the present study, GRD showed modest activity, suggesting that intracellular GSH levels are not fully depleted after exercise bouts and that the antioxidant GPX can still function efficiently. In previous reports from our laboratory, we observed that longer, moderate aerobic exercise sessions result in GRD activation [41], suggesting that active turnover of GSH as well as NADPH occurs. In addition, the increase in CAT activity after the burpee test may reinforce the antioxidant activity of GPX. CAT is an antioxidant enzyme that plays a key role in breaking down H_2_O_2_ produced by SOD in H_2_O and O_2_ [44,45]. These findings may be interpreted as follows: The burpee test seems to generate more oxidative stress than a 2000 m run in inactive, healthy individuals, as suggested by the presence of MDA together with post-exercise protein carbonyls in neutrophils and plasma. One of the candidate ROS produced in response to intense exercise is the stable and pro-oxidant molecule H_2_O_2_. For this reason, the additional CAT activity seems to enhance endogenous antioxidant activity to mitigate oxidative damage after the burpee test to a greater extent than after the 2000 m run. However, we cannot exclude other key functions of CAT, such as modulating the inflammatory response triggered after physical activity [44,45]. In this context, CAT may play a role in regulating this response and reducing cell damage. Taking this together, the activation of antioxidant enzymes after physical activity is an instrumental mechanism for maintaining cellular homeostasis and protecting against oxidative stress. However, the specific effects of this antioxidant response may depend on the type, intensity, and duration of exercise, as well as the particular conditions and health of the individual. Nevertheless, further research is necessary to better characterize the initial antioxidant response in healthy, inactive individuals when starting anaerobic training routines. This information could be instrumental in the design of antioxidant supplementation protocols.

On the other hand, MPO activation takes longer than antioxidant enzyme activation when MPO activity is determined immediately post-exercise. This particular pattern should be investigated in future research. The lack of MPO activation suggested a null immediate post-exercise inflammatory response for both types of exercise, but we cannot disregard the possibility that inflammation can develop later. On the other hand, PBMCs do not exhibit the same pattern of antioxidant enzyme activation observed in neutrophils. PBMCs are involved mainly in long-term oxidative stress, inflammation, and immunomodulation [16] but are not involved immediately after oxidative damage. This observation could explain why only CAT was activated as a rapid post-exercise antioxidant response in this cell type.

### 4.3. Novelty and Limitations of the Study

Evidence from the present report will provide information regarding timing strategies for the initiation of antioxidant supplementation in inactive individuals who decide to start high-intensity exercise routines. We speculate that intense reactions produce a strong oxidative stress response in inactive individuals, according to the low antioxidant response in PBMCs. In this situation, exogenous antioxidants could be administered during the first few days of training and then progressively reduced to allow progressive activation of antioxidant-encoding genes. This strategy could be extrapolated to sedentary overweight subjects who start to perform anaerobic exercise or adapt HIIT. This proposal is currently being studied in our laboratory.

Limitations include a lack of characterization of exercise intensity through oxygen consumption measurements. It is important to note that the sample size is small, and further studies with a larger *n* are necessary. Additionally, as suggested previously, it should be emphasized that the obtained results cannot be generalized to female populations. Moreover, students of physical activity and sports may have an adequate antioxidant system compared to other sedentary populations, so having a control group could be an interesting proposal for future research. Nevertheless, the approach presented in this report would help individuals program exercise routines as well as manage diet and recovery time.

## 5. Conclusions

In conclusion, we observed that neutrophils were the first cell type involved in the antioxidant response. SOD and GPX were activated immediately post-exercise in neutrophils. Other enzymes, such as GRD and MPO, tended to increase after the exercise bouts, although the differences from baseline were not significant. Finally, protein carbonyls appeared together with MDA as oxidative stress markers in neutrophils and plasma. The presence of more oxidative stress markers indicates that the burpee test seems to generate more oxidative stress than the 2000 m run. This finding could explain the activation of more antioxidant enzymes (SOD, CAT, and GPX) post-burpee test than post-2000 m run, where only SOD and GPX were activated.

## Figures and Tables

**Table 1:** Circulating parameters of participants at the beginning of the study (Day 1).

Parameter (Units)	Reference Values in Adult Males	Day 1
**Hemogram**		
Erythrocytes (10^6^ cells/μL)	4.4–5.9	5.3 ± 0.2
Hemoglobin (g/g serum protein)	20–26	25.4 ± 0.8
Platelets (10^3^ cells/μL)	150–450	228.1 ± 37.8
Neutrophils (10^3^ cells/μL)	1.8–7.7	3.3 ± 0.3
Lymphocytes (10^3^ cells/μL)	1.0–4.8	2.2 ± 0.4
**Circulating metabolites**		
Glucose (mg/dL)	<100	81.7 ± 5.2
Triglycerides (mg/dL)	<160	78.3 ± 8.6
Total cholesterol (mg/dL)	<200	151.6 ± 6.1
LDL (mg/dL)	<130	96 ± 22.2
HDL (mg/dL)	>55	77 ± 13.6
Creatinine (mg/dL)	0.8–1.4	0.9 ± 0.1
Uric acid (mg/dL)	2.5–8.0	4.5 ± 0.7
Urea (mg/dL)	23–44	35.3 ± 2.1
**Circulating tissue markers**		
ALT (U/L)	<40	20.9 ± 1.7
AST (U/L)	<40	22.8 ± 2.0
GGT (U/L)	<55	19.3 ± 1.8
CK (U/L)	39–308	162.5 ± 32.5

Abbreviations used: ALT, alanine aminotransferase; AST, aspartate aminotransferase; CK, creatine kinase; GGT: γ-glutamyltransferase; HDL, high-density lipoproteins; LDL, low-density lipoproteins.

**Table 2:** Changes from baseline in specific parameters after the 2000 m and burpee tests.

Parameter (Units)	Basal	Post-2000 m Test	Post-Burpee Test	d
Circulating lactate (mg/dL)	8.3 ± 1.9	26.1 ± 2.3 *	23.6 ± 3.6 ^†^	0.8
Circulating CK (U/L)	162.5 ± 32.5	181.2 ± 19.7 *	208.9 ± 22.6 ^†^	1.3
Heart rate (bpm)	58.2 ± 8.7	168.4 ± 10.2 *	171.7 ± 17.4 ^†^	0.2

After conducting ANOVA, significant differences (*p* < 0.05) were found between the post-2000 m run and the baseline (*) or between burpee tests and baseline (^†^). The effect size (d) was calculated according to Cohen.

**Table 3:** Antioxidant enzyme activities in neutrophils and PBMCs determined under basal conditions (Day 1), immediately after the 2000 m run (Day 2) and immediately after the burpee test (Day 5). MPO activity was determined only in neutrophils.

Enzymatic Activity (Units)	Basal	Post-2000 m Run	Post-Burpee Test	d
**Neutrophils**				
SOD (pkat/10^9^ cells)	22.9 ± 2.7	32.8 ± 4.6 *	28.3 ± 5.2 ^†^	0.9
CAT (k/10^9^ cells)	57.9 ± 5.1	67.5 ± 9.8	106.3 ± 14.3 ^†^	3.1
GPX (nkat/10^9^ cells)	121.1 ± 18.8	174.7 ± 21.2 *	147.5 ± 9.3 ^†^	1.7
GRD (nkat/10^9^ cells)	546.2 ± 41.3	575.4 ± 51.3	572.8 ± 26.2	0.1
MPO (µkat/10^9^ cells)	32.2 ± 6.2	33.3 ± 7.5	36.8 ± 4.9	0.6
**PBMCs**				
SOD (pkat/10^9^ cells)	51.4 ± 3.5	60.8 ± 4.2	56.1 ± 2.9	1.3
CAT (k/10^9^ cells)	89.7 ± 13.6	127.3 ± 13.6 *	113.8 ± 9.0 ^†^	1.2
GPX (nkat/10^9^ cells)	85.5 ± 9.5	97.5 ± 11.4	108.9 ± 24.0	0.6
GRD (nkat/10^9^ cells)	451.1 ± 48.6	463.4 ± 26.8	477.6 ± 25.5	0.5

After conducting ANOVA, significant differences (*p* < 0.05) were found between the post-2000 m run and the baseline (*) or between burpee tests and baseline (^†^). However, when applying the Bonferroni correction for multiple comparisons, no significant differences between post-2000 m and post-burpee test were found. The effect size (d) was calculated according to Cohen.

## Data Availability

The datasets generated during the current study are available from the corresponding author on reasonable request.

## Abbreviations

Abbreviations used: CAT, catalase; CK, creatine kinase; GPX, glutathione peroxidase; GRD, glutathione reductase; GSH, glutathione; HIIT, high-intensity interval training; MDA, malondialdehyde; MPO, myeloperoxidase; PBMCs, peripheral blood mononuclear cells; PBS, phosphate buffered saline; RONS, reactive oxygen and nitrogen species; SOD, superoxide dismutase.
